# GM-VGG-Net: A Gray Matter-Based Deep Learning Network for Autism Classification

**DOI:** 10.3390/diagnostics15111425

**Published:** 2025-06-03

**Authors:** Ebenezer Daniel, Anjalie Gulati, Shraya Saxena, Deniz Akay Urgun, Biraj Bista

**Affiliations:** 1 Department of Diagnostic Radiology, City of Hope National Medic and Center, Duarte, CA 91010, USA or ebydaniel89@gmail.com (E.D.); durgun@coh.org (D.A.U.); 2 Department of Radiology, Henry Ford Hospital, Detroit, MI 48202, USA; 3 Department of Health and Exercise Science, La Sierra University, Riverside, CA 92505, USA; saxenashraya@gmail.com

**Keywords:** deep learning, VGG Net, autism identification, ABIDE dataset, brain imaging

## Abstract

**Background:** Around 1 in 59 individuals is diagnosed with Autism Spectrum Disorder (ASD), according to CDS statistics. Conventionally, ASD has been diagnosed using functional brain regions, regions of interest, or multi-tissue-based training in artificial intelligence models. The objective of the exhibit study is to develop an efficient deep learning network for identifying ASD using structural magnetic resonance imaging (MRI)-based brain scans. **Methods:** In this work, we developed a VGG-based deep learning network capable of diagnosing autism using whole brain gray matter (GM) tissues. We trained our deep network with 132 MRI T1 images from normal controls and 140 MRI T1 images from ASD patients sourced from the Autism Brain Imaging Data Exchange (ABIDE) dataset. **Results:** The number of participants in both ASD and normal control (CN) subject groups was not statistically different (*p* = 0.23). The mean age of the CN subject group was 14.62 years (standard deviation: 4.34), and the ASD group had mean age of 14.89 years (standard deviation: 4.29). Our deep learning model accomplished a training accuracy of 97% and a validation accuracy of 96% over 50 epochs without overfitting. **Conclusions:** To the best of our knowledge, this is the first study to use GM tissue alone for diagnosing ASD using VGG-Net.

## 1. Introduction

Autism Spectrum Disorder (ASD) is a progressive condition characterized by difficulties in social interaction, communication, stereotypic behaviors, and sensory abnormalities [1,2]. In the field of medical imaging, deep learning models have made significant strides in ASD diagnosis, leveraging their unsupervised nature to identify complex patterns [3,4].

Magnetic resonance imaging (MRI)-based studies have shown various biomarkers that demonstrated altered patterns of gray matter in autism patients compared to the normal control population [5,6]. For example, increased gray matter has been reported in angular gyrus in the right hemisphere, the prefrontal cortex, superior and middle frontal gyri in the left hemisphere, the precuneus and inferior occipital gyrus in the left hemisphere and the inferior temporal gyrus in the right hemisphere regions as increased biomarkers in autism subjects. In addition to these increased gray matter tissues in the brain, they also reported diminished gray matter tissues in the left hemisphere post central gyrus and cerebellar regions [7]. Another study compared three groups, normal control (CN), participants with attention deficit hyperactivity disorder (ADHD), and ASD, and found that gray matter volume (GMV) was significantly higher in the ASD group compared to the ADHD and CN groups (*p* = 0.004). Total brain volume (TBV) was also significantly higher in the ASD group (*p* = 0.015) [8]. Another longitudinal volumetric study with 156 participants also exhibited statistically significant increases in GMV and TBV in ASD subjects [9]. Even though the sample sizes in the previous study were relatively small (33 CN, 44 ADHD, and 19 ASD), the observed group differences in GMV and TBV were statistically validated using Bonferroni correction [8]. These findings might be an indication that gray matter tissue alone could potentially serve as a useful biomarker for classifying ASD through deep learning approaches.

Another longitudinal volumetric study with 156 subjects also reported a similar pattern of statistically significant increased gray matter and volume in ASD subjects [9]. Although, the sample sizes were small for CN, ADHD, and ASD subjects, such as 33, 44, and 19, respectively. Nonetheless, the results of the GMV and TBV group differences were based on Bonferroni statistical correction [8], suggesting that these results might indicate the potential for further classification of ASD brain patterns using gray matter tissues alone over a deep learning approach. Another study consisted of 295 study cohorts that studied MRI-based brain changes associated with ASD, with respect to gender differences. The study noted that males with ASD displayed increased gray matter volumes in the insula and superior frontal gyrus, while diminished volumes were noted in the inferior frontal gyrus and thalamus. However, females with ASD exhibited increased gray matter volume in the right cuneus [10]. In addition to gray matter biomarkers, other works have reported on white matter changes, including white matter connectivity, which also provide a significant biomarker for ASD [11,12,13,14]. For example, a diffusion tensor imaging (DTI)-based work reported a 99% classification accuracy for ASD using fivefold cross validation [15].

Previous studies have used various deep learning approaches to identify ASD based on functional MRI (fMRI) and structural MRI (sMRI) data, showing a wide range of classification accuracies. A study using a 3D Residual Network (ResNet-18) and multilayer perceptron (MLP) achieved 74% accuracy using fMRI and region of interest (ROI) data [16]. Another study using a deep neural network (DNN) approach to fMRI data reported a 70% classification accuracy, with ROIs selected based on co-activation levels of brain regions [17]. Another hybrid model that combined fMRI and structural MRI data, including gray and white matter tissues, for a Deep Belief Network (DBN) approach accomplished 65% accuracy, with 116 ROIs used from both imaging modalities [18]. A connectivity-based study using 7266 gray matter ROIs from the Blood Oxygen Level Dependent (BOLD) signal tested on 964 subjects from the Autism Brain Imaging Data Exchange (ABIDE) dataset achieved a 60% classification accuracy [19].

Another study reported improved accuracy with smaller sample sizes, such as a study with 80 subjects using a leave-one-out classifier that achieved 79% accuracy, which boosted to 89% for subjects under 20 years of age [20]. A DNN classifier on fMRI data involving 866 subjects (402 ASD and 464 control subjects) showed a high classification accuracy of 88%, using ROIs based on several functional and structural atlases, including the Bootstrap Analysis of Stable Clusters (BASC) and the Craddock 200 (CC200) atlas [21]. A convolutional neural network (CNN) approach, using 126 subjects from the ABIDE database, achieved an impressive 99.39% accuracy over 50 epochs with 20% of the data reserved for validation [22]. Additionally, a multimodal fusion approach incorporating both fMRI and sMRI for 1383 male participants aged 5 to 40 years achieved an accuracy of 85%, with the structural model alone achieving 75% and the functional model achieving 83% [23]. These findings highlight the effectiveness of different deep learning models and imaging modalities in ASD classification, with multimodal approaches offering the highest accuracies.

While fMRI provides valuable physiological information about brain regions, it has lower resolution and more attenuation of structural regions. In contrast, sMRI offers higher resolution and less attenuation of structural regions, making it a promising tool for studying brain anatomy. However, its application in ASD prediction using deep learning models has been relatively underexplored. In this work, we aim to use sMRI images alone to train and predict outcomes in a deep learning model. For this purpose, we used the VGG network, introduced by Simonyan and Zisserman in 2014 for the ImageNet Challenges. The VGG network has proven effective in large image data challenges, particularly in image recognition [24]. Previously, a study used VGG16 model to identify papillary thyroid carcinoma from benign thyroid nodules using cytological images, achieving 97.66% accuracy in cancer detection [25].

In our study, we introduce a modified VGG model, leveraging the strengths of the base VGG model along with multiple weighted layers in a deep neural network, using TensorFlow and Keras, to improve ASD identification in large datasets. To the best of our knowledge, this is the first study to apply the VGG model for ASD identification based solely on sMRI. While the majority of conventional deep learning models in the literature have used multimodal or multiple tissue types for ASD classification, our approach focuses on identifying ASD using GMD maps alone, minimizing computational complexity in terms of storage and learning.

## 2. Materials and Methods

### 2.1. Dataset

The present study utilized MRI T1-weighted image data from the ABIDE database. ABIDE is a consortium that provides previously collected sMRI and rs-fMRI data from individuals with ASD and normal controls for data sharing within the scientific community [26]. We included a total of 272 subjects in our analysis and the age difference between the ASD and CN groups was assessed using an independent *t*-test from the SciPy Python 3.8 library, implemented within the PyCharm platform. 

### 2.2. Preprocessing of MRI-T1 Images

For data preprocessing, we employed the statistical parametric mapping package SPM12 (Wellcome Department of Cognitive Neurology, London, UK) and MATLAB 2019.b (The MathWorks Inc., Natick, MA, USA) with custom software to preprocess our MRI T1 images. The preprocessing steps followed those described earlier [27]. The Diffeomorphic Anatomical Registration Through Exponentiated Lie Algebra (DARTEL) toolbox was used to improve inter-subject image registration in our input images [28]. We segmented gray matter (GM), white matter (WM), cerebrospinal fluid (CSF), skull, and other brain regions using the ‘new segment’ option in the DARTEL toolbox. The gray matter probability maps computed for each scan were spatially normalized to Montreal Neurological Institute (MNI) space (unmodulated, re-sliced to 1 × 1 × 1 mm) and smoothed with a Gaussian filter (9 mm full width at half maximum) [29,30,31,32].

### 2.3. Conventional VGG-16 Architecture

In the conventional VGG-16 network, convolutional layers are followed by pooling layers in each hidden layer unit. The deep learning network starts with 64 filters in the first layer unit, then to 128 filters, then 256 filters, and finally obtains 512 filters in the deeper hidden layers. Furthermore, each convolutional layer utilities a rectified linear unit (ReLU) for activation. Finally, it incorporates three fully connected layers: the first two with 4096 channels, and the third with 1000 channels—one for every class.

### 2.4. Proposed Deep Learning GM-VGG-Net Architecture

**Figure 1:**
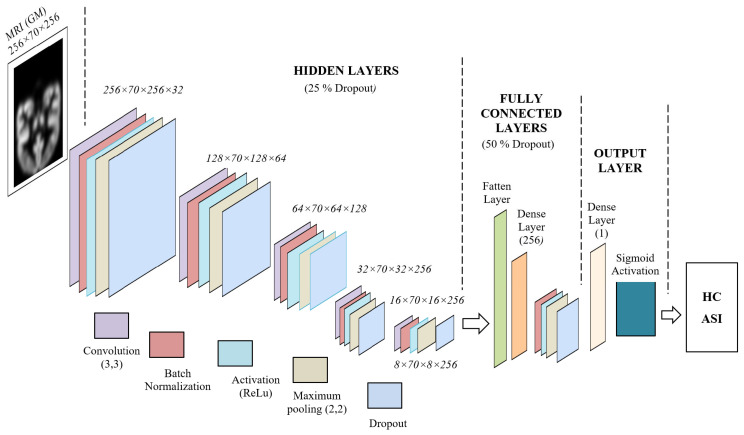
Proposed deep learning VGG network for ASD and HC classification.

The proposed deep learning network is implemented using the TensorFlow 1.4 and Keras platforms. Skull-stripped image data (segmented and normalized) provided a higher probability of achieving valid MNI coordinates for functional activations compared to skull-included input images [33]. Our deep learning architecture starts with 32 filters, followed by 64 and 128 filters, and ends with two final units, each containing 256 filters. We have also encompassed further batch normalization units, as demonstrated below. The proposed deep learning VGG network architecture is shown in Figure 1. Nevertheless, we have significantly changed the filter layout and layer structure in our version of this deep neural network [24,34,35,36].

#### 2.4.1. Input Layer Unit 

The input layer consists of preprocessed gray matter (GM) from MRI T1-weighted images. To reduce complexity, we selected the best 70 slices that contain the most brain regions (256 × 70 × 256), where 256 represents the image dimensions and 70 is the number of selected slices. Our network was designed with five fully connected, sequential hidden layer units. Each hidden layer unit was designed with the following layers: convolution filters (3 × 3), activation unit (ReLU), maximum pooling layer (2 × 2), and 25 percentage of dropout layers.

#### 2.4.2. Hidden Layer Unit

The first hidden layer consists of 32 convolution filters with a kernel size of 3 × 3. The output of the convolution layer in the first hidden layer has 32 feature maps. The maximum pooling layer of the first hidden layer reduces the dimensionality of the feature map by half, i.e., 128 × 70 × 128 × 32 feature maps. The second hidden layer unit consists of 64 convolution filters with a kernel size of 3 × 3, and the output of the convolution layer in the second hidden layer unit has 64 feature maps. The maximum pooling layer of the second hidden layer reduces the dimensionality of the feature map by half, i.e., 64 × 70 × 64 × 64 feature maps. The third hidden layer unit is designed with 128 convolution filters with a kernel size of 3 × 3, and the output of the convolution layer in the third hidden layer unit has 128 feature maps. The maximum pooling layer of the third hidden layer reduces the dimensionality of the feature map by half, i.e., 32 × 70 × 32 × 128 feature maps. The fourth hidden layer unit is developed using 256 convolution filters with a kernel size of 3 × 3, and the output of the convolution layer in the fourth hidden layer unit has 256 feature maps. The maximum pooling layer of the fourth hidden layer reduces the dimensionality of the feature map by half, i.e., 16 × 70 × 16 × 256 feature maps. The fifth (final) hidden layer unit is implemented with 256 convolution filters with a kernel size of 3 × 3. The maximum pooling layer of the fifth hidden layer reduces the dimensionality of the feature map by half, i.e., 8 × 70 × 8 × 256 feature maps. In our proposed deep learning network, each hidden layer unit is designed with a rectified linear unit (ReLU)-based activation, a dropout layer with 25%, and batch normalization functions.

#### 2.4.3. Fully Connected Layer Unit

Our proposed fully connected (FC) layer unit is designed with a flatten layer, a fully connected layer, a batch normalization layer, a ReLU-based activation, a maximum pooling layer, and a 50% dropout layer. The FC layer connects the hidden layers to the output layer unit.

#### 2.4.4. Output Layer Unit

The output layer unit is designed with a dense layer and a sigmoid activation function. The output unit predicts our images into ASI and HC classes.

## 3. Results

### 3.1. Demographic Data

We included a total of 272 subjects, with 132 individuals diagnosed with ASD and 140 matched normal controls. The mean age of the CN group was 14.62 years (SD = 4.34), and the mean age of the ASD group was 14.89 years (SD = 4.29). The CN group consisted of 68 male subjects and 72 female subjects, while the ASD group consisted of 67 males and 65 females. The mean age of males in the CN group was 14.97 years (SD = 4.14), and in the ASD group, it was 15.75 years (SD = 3.77). The mean age of females in the CN group was 13.57 years (SD = 4.56), and in the ASD group, it was 14.02 years (SD = 4.60). No significant age differences were observed between the two groups (*p* = 0.23), as shown in Table 1.

### 3.2. Performance Evaluation of GM-VGG Net Classifier

**Figure 2:**
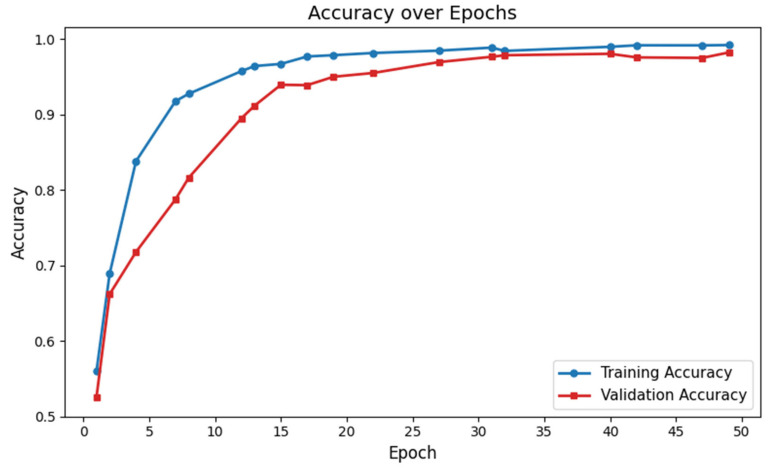
Training and validation accuracy of the proposed deep learning network for ASD identification.

The classification performance of our proposed GM-VGGNet was evaluated based on loss and accuracy parameters. The training and validation loss functions, along with the accuracy of our deep network, are shown in Figure 2. Our proposed deep learning network was validated over 50 epochs. The training and validation accuracy of our network were 97% and 96%, respectively, over 50 epochs. The loss function values of our network were 0.0204 for training and 0.0696 for validation over 50 epochs. In this deep learning model, we used the TensorFlow–Keras platform with the Adam optimizer, kept at the default learning rate of 0.001. We fine-tuned the structure based on the loss and accuracy performance to avoid overfitting challenges. In this work, the image dataset was split into 70% for training and 30% for validation. The total number of parameters was 5,176,705, of which 5,174,721 were trainable. The model summary is given in Table 2.

## 4. Discussion

In this study, we developed a deep learning network for ASD identification, utilizing only structural GM tissue images and based on the VGG16 architecture. We systematically evaluated the network’s performance and achieved the highest accuracy on sMRI data after 50 epochs of training. To the best of our knowledge, the proposed deep learning model outperformed exciting network for ASD identification using structural GM tissues alone. By using the deep learning network with GM maps exclusively, we were able to reduce the complexity of the training process.

Previous studies on ASD classification using the ABIDE dataset have reported a classification accuracy of 63.89% for gray matter (GM) tissue alone, using a tenfold cross-validation with a DBN [18]. The classification accuracy was improved to 65% when fMRI data alone, along with GM tissue, were used. Finally, by combining features from white matter (WM) tissues, GM, and fMRI, they achieved an accuracy of 65.56% for ASD classification. Our model demonstrates superior performance in classification compared to the previous model [18]. We tested our model on a dataset of 272 samples, whereas their model was evaluated on 185 data samples. In their previous work, the fMRI-based model showed lower performance than the sMRI GM tissue alone images, possibly due to the low temporal resolution from the hemodynamic response, as well as susceptibility artifacts from signal dropout [37]. Therefore, our GM-VGG16 model has less computational complexity while maintaining greater accuracy, as it relies solely on GM tissues.

Another study on male participants from the ABIDE dataset, which incorporated 1383 subjects, exhibited higher performance with an fMRI model compared to sMRI. Their accuracy reached 75% with sMRI alone, while fMRI achieved 83%, and the combined fused data reached an accuracy of 85%. The previously reported model [18] performed differently with lower accuracy, possibly due to the high spatial resolution of their method, or due to the fusion approach they employed, which used early fusion to combine sMRI and fMRI before classification. In contrast, later fusion approaches integrate features based on the classification performance during label testing. However, their feature extraction models required more manual involvement, leading to a semi-automated approach. Our method, on the other hand, does not rely on feature selection from the images; instead, our model is trained to identify ASD patterns directly from the whole GM maps.

The architecture of our deep learning network is based on the VGG network, which was developed by Karen Simonyan and Andrew Zisserman for the ImageNet Challenge in 2014 [24]. The conventional VGG addressed the challenges of training deep neural networks for large scale image recognition, reaching higher accuracy. Furthermore, VGG16 has demonstrated 97.66% accuracy on cytological images for papillary thyroid carcinomas [25]. Similarly, we employed small 3 × 3 convolution filters for feature map generation. Our network consists of five sequential hidden layers, each with 2 × 2 max pooling and a stride of 2. As with the VGG architecture, the width of the convolution filters increases sequentially across all hidden layers, starting with 32 filters in the first hidden layer and progressing to 256 filters in the final hidden layer. Unlike conventional neural networks, which typically use smaller input sizes (e.g., 32 × 32 pixels), the VGG network is designed to handle larger input sizes effectively [24,25]. Larger input sizes preserve more substantial brain regions, generating more active feature maps.

**Figure 3:**
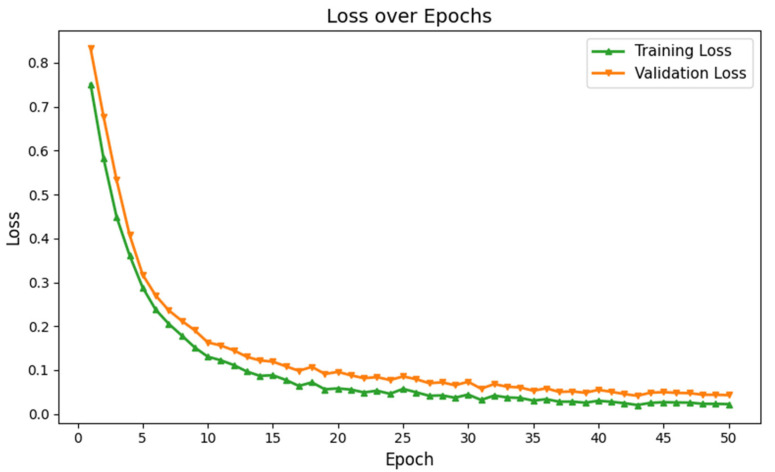
Training and validation loss of the proposed deep learning network for ASD identification.

However, unlike the original VGG network, we incorporated batch normalization across all five hidden layers, which improved training accuracy. Each hidden layer in our network uses a rectified linear unit (ReLU) activation function, and we applied a uniform 25% dropout rate to prevent overfitting. This dropout rate was fixed using the trial and error, as the network showed poor learning without it, and started to memorize the training data. Our deep learning architecture showed a lower error difference between training and validation over 50 epochs, as shown in Figure 3. These results exhibit that our network overcame the overfitting limitations and enabled higher learning.

In contrast to the conventional VGG network, which includes three fully connected layers and a dropout layer with a 0.5 rate [24], our network features a fully connected (FC) unit and an output layer (OL). In our work, the FC unit was designed with a flatten layer, a dense layer (256 filters), a batch normalization layer, activation layer, and a 50 percentage of dropout layer. Moreover, in our output layer, we used a sigmoid activation, while the conventional VGG network utilizes softmax activation. Our proposed gray matter-based deep learning network has a higher performance in terms of validation accuracy and loss function over the various existing ASD identification models.

However, there are some limitations in our study. Our deep learning network was trained solely on the ABIDE dataset, and future work should involve incorporating additional datasets to further validate our approach. Furthermore, our model was tested on 272 MRI images, and a larger dataset is needed to enhance the model’s generalization. Although our model does not require feature extraction during training and validation, our preprocessing, which involved segmenting the GM tissues, was performed semi-automatically using the SPM 12 toolbox. In the future, a fully automated approach for GM tissue segmentation should be integrated into the deep learning network, alongside the classification model. Moreover, we acknowledge that automated hyperparameter optimization approaches were not applied in this pilot work. While we used standard default settings and fine-tuned network structure based on observed performance, future work will include efficient optimization techniques to increase the efficiency. Additionally, this exploratory pilot work was focused on evaluating the proposed GM-VGG-Net using validation and training accuracy and error measures obtained via TensorBoard with Keras. Although these metrics offer a useful baseline, future work will combine more broad evaluation measures such as F1-score, AUC-ROC, and confusion matrices. We also plan to benchmark the model against recent state-of-the-art architectures using the largest dataset to provide more rigorous comparative analysis. Despite these challenges, our model achieved the highest performance, minimizing classification loss over 50 epochs.

## 5. Conclusions

The developed deep learning network, which is a modified VGG architecture named the gray matter network (GM-VGG-Net), demonstrates an effective method for classifying ASD using sMRI brain scans based exclusively on gray matter (GM) tissues. Our modified GM-VGG-Net showed a training accuracy of 97% and a validation accuracy of 96% over 50 epochs. This methodology is significant as it based on sMRI GM maps, which streamlines the training process, reduces computational complexity, and outperforms previous models that required multimodality or whole brain data.

## Figures and Tables

**Table 1:** Demographic data.

Variable	CN	ASD
*N*	140	132
Age (mean ± SD)	14.62 ± 4.34	14.89 ± 4.29 (*p* = 0.23)
Age (male) (mean ± SD)	14.97 ± 4.14	15.75 ± 3.77
*N* (male)	68	67
Age (female) (mean ± SD)	13.57 ± 4.56	14.02 ± 4.60
*N* (female)	72	65

Abbreviations: CN (control group), ASD (Autism Spectrum Disorder), SD (standard deviation), and *N* (number of subjects). The *p*-value was tested between the groups for age and is considered significant at a threshold of 0.05. The total number of subjects is 272.

**Table 2:** Model summary of proposed deep learning network.

Parameter	Value
Optimizer	Adam
Learning Rate	0.001
Epochs	50
Trainable Parameters	5,174,721
Non-Trainable Parameters	1984
Total Parameters	5,176,705

## Data Availability

The data for this study were obtained from the ABIDE database, which operates under defined accessibility protocols as outlined by the source.
